# Revisiting Nonlinear Functional Brain Co-activations: Directed, Dynamic, and Delayed

**DOI:** 10.3389/fnins.2021.700171

**Published:** 2021-10-12

**Authors:** Ignacio Cifre, Maria T. Miller Flores, Lucia Penalba, Jeremi K. Ochab, Dante R. Chialvo

**Affiliations:** ^1^Facultat de Psicologia, Ciències de l'Educació i de l'Esport, Blanquerna, Universitat Ramon Llull, Barcelona, Spain; ^2^Center for Complex Systems and Brain Sciences (CEMSC^3^), Escuela de Ciencia y Tecnología, Universidad Nacional de San Martín, Buenos Aires, Argentina; ^3^Institute of Theoretical Physics and Mark Kac Center for Complex Systems Research, Jagiellonian University, Krakow, Poland; ^4^Consejo Nacional de Investigaciones Científicas y Tecnológicas (CONICET), Buenos Aires, Argentina

**Keywords:** fMRI, resting state networks, functional connectivity, dynamic functional connectivity, autism (ASD)

## Abstract

The center stage of neuro-imaging is currently occupied by studies of functional correlations between brain regions. These correlations define the brain functional networks, which are the most frequently used framework to represent and interpret a variety of experimental findings. In the previous study, we first demonstrated that the relatively stronger blood oxygenated level dependent (BOLD) activations contain most of the information relevant to understand functional connectivity, and subsequent work confirmed that a large compression of the original signals can be obtained without significant loss of information. In this study, we revisit the correlation properties of these epochs to define a measure of nonlinear dynamic directed functional connectivity (*nldFC*) across regions of interest. We show that the proposed metric provides at once, without extensive numerical complications, *directed* information of the functional correlations, as well as a measure of *temporal lags* across regions, overall offering a different and complementary perspective in the analysis of brain co-activation patterns. In this study, we provide further details for the computations of these measures and for a proof of concept based on replicating existing results from an Autistic Syndrome database, and discuss the main features and advantages of the proposed strategy for the study of brain functional correlations.

## 1. Introduction

The large scale dynamics of the brain exhibits a plethora of spatio-temporal patterns. Since the first description of voxel-wise correlation networks (Eguíluz et al., 2005), there has been a continuous interest in developing better ways to derive brain “networks” from fMRI time series data. Common to all is the identification of functional “nodes” [i.e., fMRI time series extracted from regions of interest (ROI)], functional edges (i.e., the cross-correlations), which allows for the subsequent graph analysis. An important methodological challenge has been always to define an adequate coarse graining of the brain imaging data to compress 1,000 of the so-called blood oxygenated level dependent time series. The usual analysis aims at the identification of bursts of correlated activity across certain regions, which requires extensive computations, complicated in part by the humongous size of the data sets.

In the previous study, we proposed that the timing of the brief epochs of relatively stronger BOLD activations contain a great deal of functional connectivity (FC) information (Tagliazucchi et al., 2011, 2012). The results of subsequent work (Liu and Duyn, 2013; Liu et al., 2013; Petridou et al., 2013; Wu et al., 2013; Amico et al., 2014; Jiang et al., 2014; Li et al., 2014; Allan et al., 2015; Chen et al., 2015; Tagliazucchi et al., 2016) seems to provide ample support to this idea, by confirming the functional relevance of such relatively large amplitude BOLD events under a variety of conditions.

The is study goes beyond the analysis of correlations between BOLD time series to explore and define a set of measures of the *nonlinear directed dynamic functional correlation* across ROIs. The use of such measures, despite its simplicity, may help to expand at once the perspective of the usual FC paradigms, such as seed correlation maps and networks, into the realms of nonlinear time-dependent directed correlations.

The study is organized as follows: In the next section, we describe the essence of the method, starting with the basic procedure to define the BOLD-triggered events followed by a description of the available correlation measures that allow a proper definition of the functional connectivity between the events, including a definition of directionality and temporal lag of the events. Section 3 contains the analysis of a simple example as a proof of concept of healthy subject fMRI data set, followed by the replication and further analysis of a voxel-wise published data set from Autism Syndrome in order to show the method features. This study closes with a discussion of the advantages and limitations of the method and potential implications of the results. Derivations and further technical details are condensed in the Supplementary Material.

## 2. Methods

The analysis to be discussed can use BOLD time series recorded indistinctly from either resting state conditions or during an experiment in which the subject is performing a given task. The most common approach to determine functional connectivity is to compute Pearson's linear correlation between BOLD time series (van den Heuvel and Hulshoff P., 2010; Finn et al., 2015). In contrast, the objective of the present analysis is to determine the relation between relatively large amplitude BOLD activations from a given pair of signals. In this section, it will be discussed: 2.1 how large amplitude events are selected given series of fMRI data; 2.2 correlations computed with the selected events; 2.3 how directionality is understood when working with events; and 2.4 how the dynamic connectivity, understood here as lags between time series, is computed.

### 2.1. Definition of BOLD-Triggered Events

**Figure 1:**
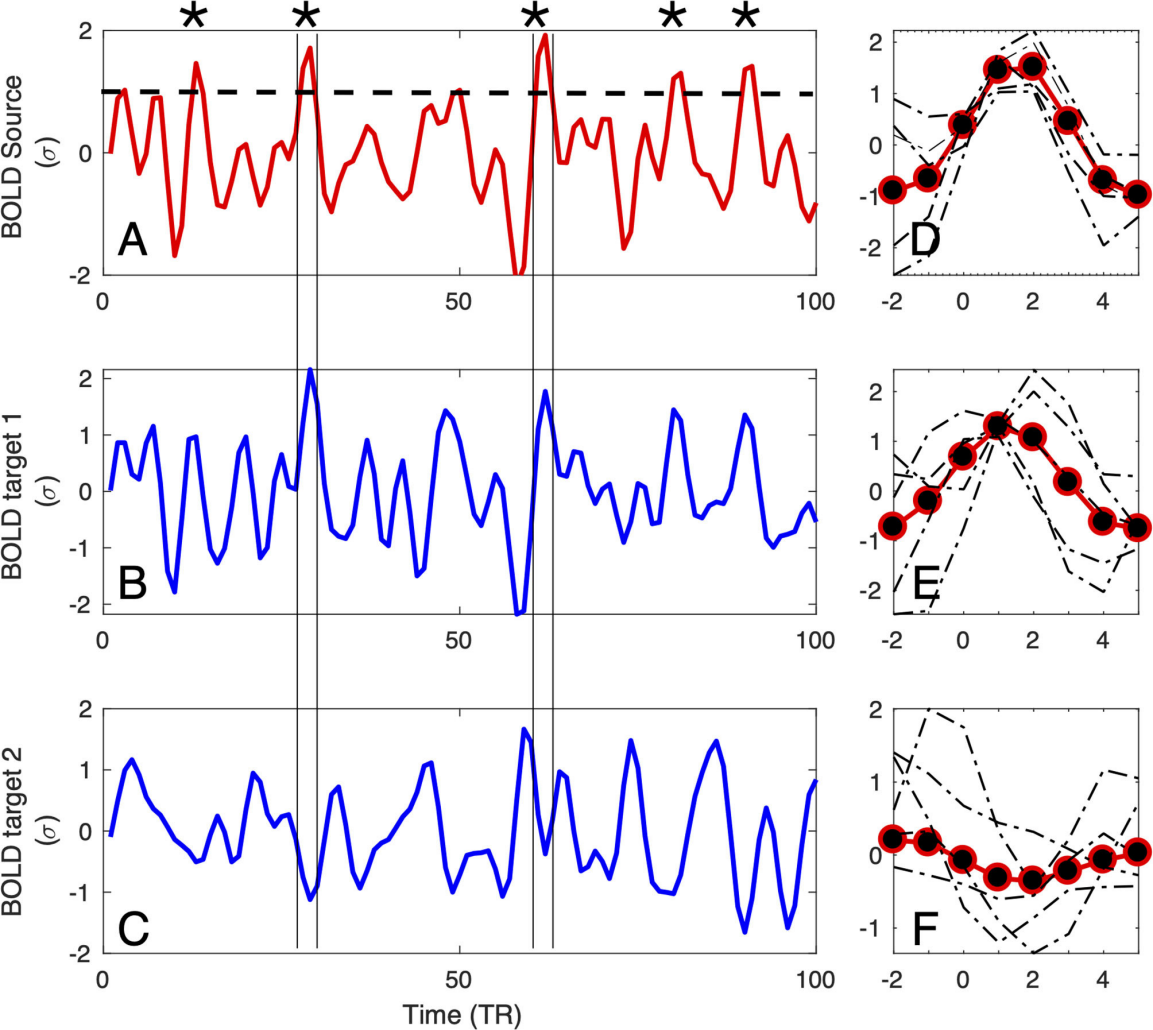
Definition of the large amplitude BOLD events: For each source region **(A)**, BOLD-triggered events (asterisks) are defined at the times at which the BOLD signal crosses an arbitrary threshold [here set to 1σ, denoted by the dashed line in **(A)**]. For each detected source event, a target event is extracted (coinciding with the times of the source) from the BOLD signals of the other regions of interest (as the two examples in **(B,C)** denoted by vertical lines). Subsequently, the extracted events can be averaged **(D–F)**, and used for further computation of correlations, delays, and directionality.

First, each BOLD time series is z-scored (its mean is subtracted, and it is divided by its SD). Next, a threshold for detecting strong activity is chosen, (typically the results remain unchanged when using a range of 1 − 2 SDs) and for each time series, the timing of each upward threshold crossing is determined (Figure 1A). Note that the number of threshold crossings depends on the auto-correlation of the BOLD signals (which stays in the range 0.6–0.85 Ochab et al., 2019) and more generally on the exponent of the 1/*f*^α^ frequency spectrum. Empirically, for the threshold of 1σ, in a BOLD signal we find on average 8.5 ± 2.8 upward crossings per 4 min of fMRI scan.

The timing is further used to define the *seed* or *source* events. For a given seed voxel or region of interest (ROI), they consist of segments of BOLD time series starting typically 4 − 5 *s* before and ending 9 − 15*s* after the crossing (which translates to 2 − 3 TRs before and 4 − 7 TRs after, with *TR* = 2.3 in the data we are using as a proof of concept in this study). This timescale is chosen by the typical duration of these events, which in turn is dictated by the longest timescale of the hemodynamic response function (~10 − 15 s).

Finally, for each seed event, the *target* events are extracted from all the other BOLD time series at the exact same times as the seed, see Figures 1B,C. The average time courses of the events follow typically a smooth pattern, although they do exhibit variability, for both the seed (see Figure 1D) and targets (see Figures 1E,F). If the interest of a given experiment is to define an average inter-relation measure between ROIs, then all the seed and target events can be averaged (as shown by red-and-black circles in Figures 1D–F), for instance over the entire scan fMRI session.

### 2.2. Correlations

Once the source and the target events are extracted from the BOLD time series, a few options of computing correlations are possible:

1. *r*_*P*_(*i, j*) linear Pearson's correlation between the whole time series *i* and the whole time series *j* (computed in section III where we perform a proof of concept). This option is not related to events, but in the next section we will compute for comparison purposes,
2. $r_{E}^{\left(k\right)}\left(i,j\right)$ linear correlation between a *k*-th source event in time series *i* and a respective target event in time series *j*. This option seems the most plausible when analyzing transient events, for instance localized tics on a motor disease.
3. ${\bar{r}}_{E}\left(i,j\right)=1/K\overset{K}{\underset{k=1}{\sum }}r_{E}^{\left(k\right)}\left(i,j\right)$ average linear correlation between *K* source events in time series *i* and respective target events in time series *j*,
4. *r*_*C*_(*i, j*) linear correlation between concatenated source events in time series *i* and concatenated respective target event in time series *j*,
5. *r*_*E*_(*i, j*) linear correlation between an average source event in time series *i* and an average target event in time series *j* (computed in section III where we perform a proof of concept).

In this study, we will only use measures defined by 1 and 5. The other choices, 3, 4, are not discussed here, but it is worth considering them in future studies to obtain statistically less biased estimators of correlations.

### 2.3. Directionality

**Figure 2:**
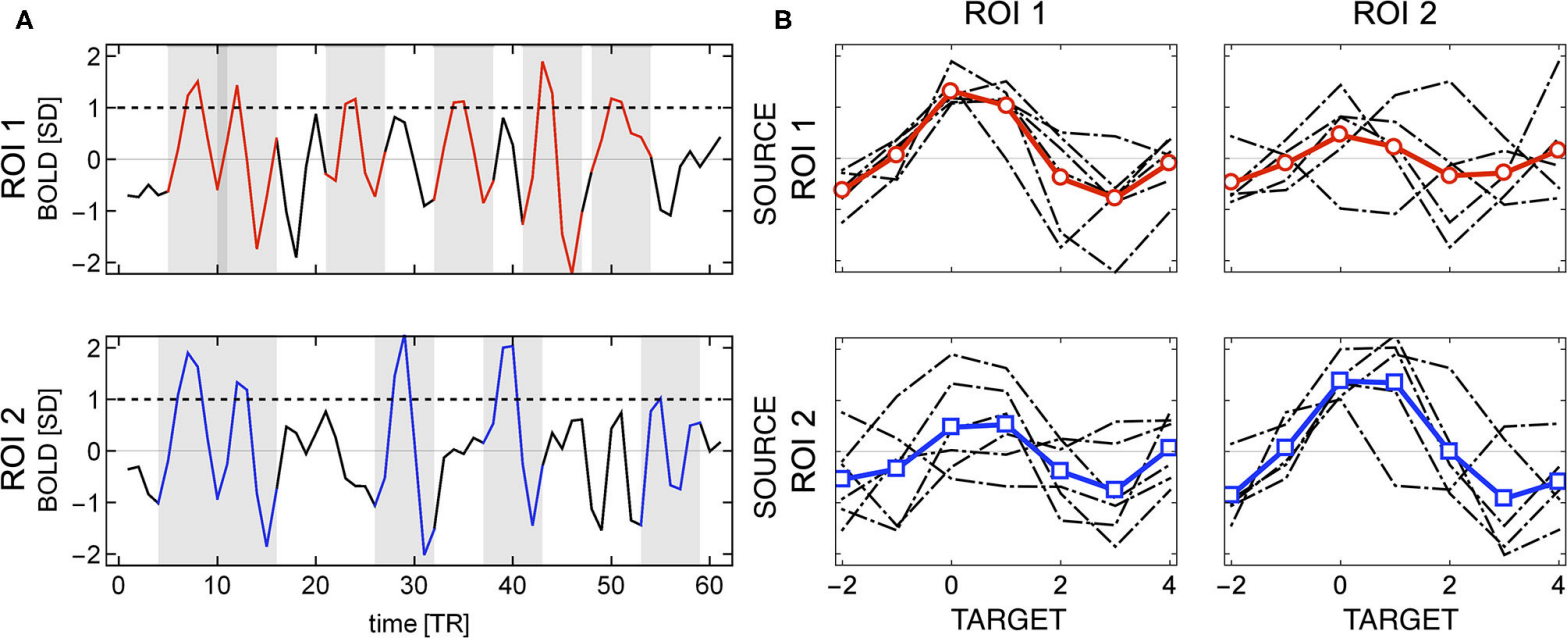
Example of directionality in source events of two regions of interest. **(A)** The shaded areas indicate the location of the source events (BOLD activity around threshold crossing). The source events of ROI 2 may appear at different times than source events of ROI 1 (e.g., around *TR* = 30 and *TR* = 40). **(B)** Individual events (in gray) and their averages (in red for ROI 1 and blue for ROI 2); source events are shown in the diagonal subplots, and target events in the off-diagonal ones. Different sets of source events for each ROI give rise to asymmetry in the correlations between any two regions.

Given two regions of interest *i* and *j*, the linear Pearson correlation between their BOLD time series by definition is symmetric, i.e., *r*_*P*_(*i, j*) = *r*_*P*_(*j, i*). It is not the case, if the correlations are computed using events. Then, the distinction between source and target becomes relevant, as shown in Figure 2A. The shaded areas in the plots mark the positions of source events of each of the two relatively strongly correlated ROIs. Visibly, the first two events are common for both time series, but for instance the BOLD activations around *TR* = 30 and *TR* = 40 are source events for ROI 2 but not for ROI 1.

Consequently, the set of events over which one computes correlations when ROI 1 is considered the source is different from those observed when ROI 2 is considered the source, as it can be seen in Figure 2B. The four plots in Figure 2B, shows an example for two ROI's in which (in a matrix format) the sources as columns and the targets as rows. The top left panels contain the source events of ROI 1 (and its average) and the top right one its target (ROI 2). Similarly, the bottom right panel shows the source events extracted from ROI 2 (and its average) and the left bottom one its target (ROI 1). So even though the BOLD series of both regions are highly correlated, the source and target events are different, and hence, the event correlation is not symmetric *r*_*E*_(*i, j*) ≠ *r*_*E*_(*j, i*).

The asymmetry in the correlations may indicate that on average, the co-activations between regions have a preferred direction. Being cautious about extrapolating these results to neuronal activation, we can estimate and assess a global correlation asymmetry of the functional connectivity by computing

(1) $$\begin{array}{r} A=\underset{i,j}{\sum }\left(r_{E}\left(i,j\right)-r_{E}\left(j,i\right)\right), \end{array}$$

for a given region, or similarly to determine the asymmetry of each ROI, or of each pair of time series *i* and *j*. In practice, we computed this metric subtracting the transposed mean correlation matrix from the non-transposed one (see Supplementary Material 1).

The directionality can be also computed , in the spirit of analysis of point processes (Tagliazucchi et al., 2012, 2016; Cifre et al., 2020), from the relative number of events occurring simultaneously in two regions. For instance, in Figure 2, there are two out of six source events in ROI 1 that are also triggers (i.e., above threshold) in ROI 2, and two out of five in ROI 2 that are also triggers in ROI 1. This approach takes into account event amplitudes, which to a large extent could be also achieved by computing covariance instead of the Pearson correlation between source and target events. Below, we call such ratio event directionality.

### 2.4. Delays

Several studies (Mitra et al., 2014, 2015a,b; Mitra and Raichle, 2016, 2018) have provided consistent evidence for the presence of very slow (>1 s) fluctuations in the fMRI BOLD signal propagating throughout the neocortex, thalamus, striatum, and cerebellum. More recently, these slow waves of activity were shown to be associated with spontaneous arousal fluctuations that, in turn, can account for the topographic organization of the brain functional connectivity (Raut et al., 2021). This information was gathered by the use of conventional lagged cross-covariance between pairs of BOLD time series *x*_*i*_(*t*) and *x*_*j*_(*t*) extracted from regions *i* and *j*:

(2) $$\begin{array}{r} {\text{C}}_{i,j}\left(\tau \right)=\frac{1}{T}\overset{T}{\underset{t=1}{\sum }}x_{i}\left(t+\tau \right)x_{j}\left(t\right) \end{array}$$

where τ is the lag (in units of TRs). The value of τ(*i, j*) at which C_*i,j*_(τ) exhibits an extremum defines the delay between signals *x*_*i*_ and *x*_*j*_. To improve the resolution beyond multiple integers of TR, a parabolic interpolation of the cross-covariance extremum allows to determine the temporal lags with a finer resolution, as done in Mitra et al. (2014). Since by definition the time delay matrix τ(*i, j*) is anti-symmetric, i.e., τ(*i, j*) = −τ(*j, i*), the information on the cross-covariance value and the lags can be used to determine the structure of the entire spatio-temporal processes.

**Figure 3:**
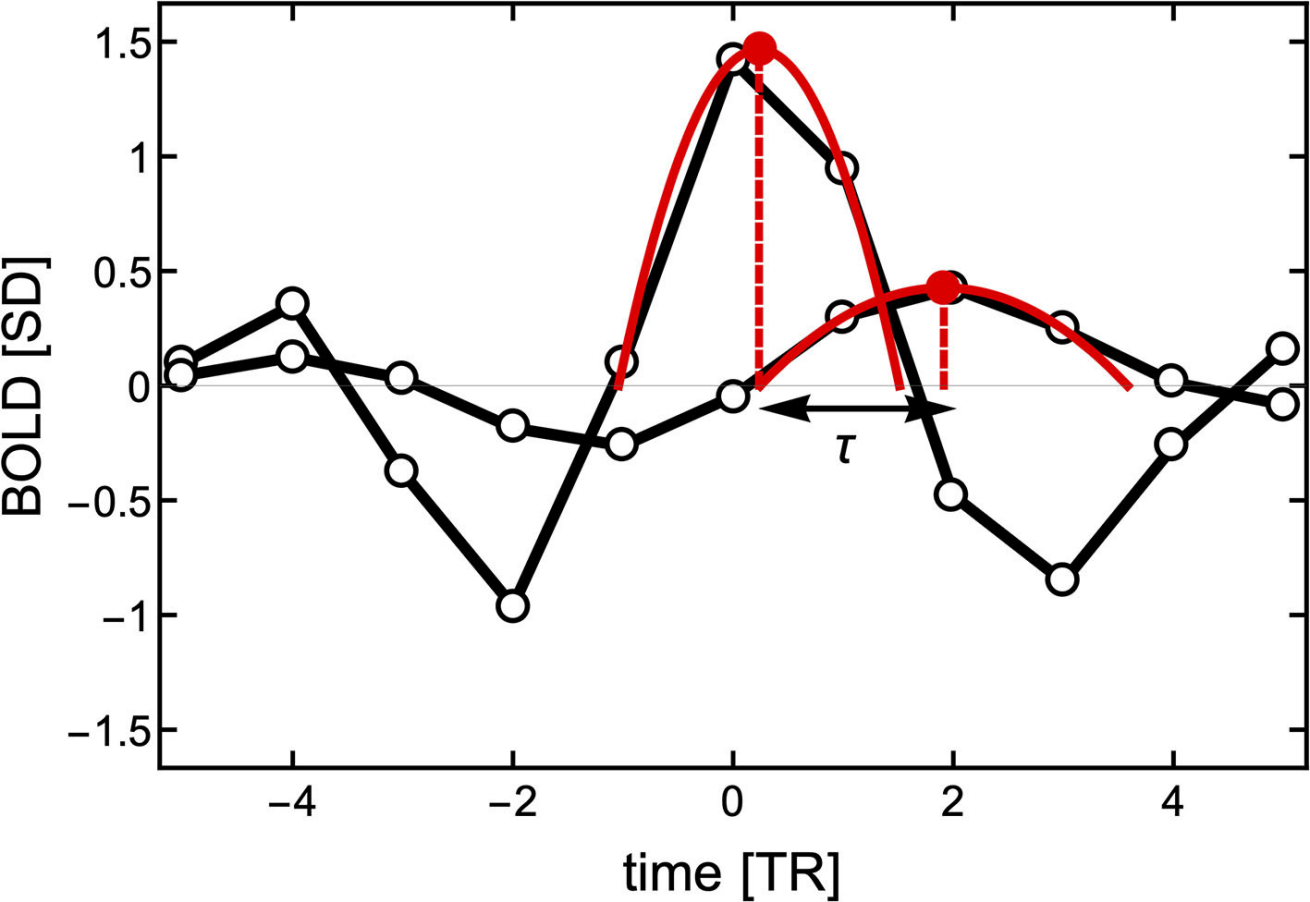
Estimation of the delay τ between two events with finer resolution than the TR. First, the peak of the source event is centered at time *TR* = 0 to estimate the closest peak of the target signal (here around time *TR* = 2). To obtain a better resolution of the delay between the two signals, two parabolas are fitted to three points in each of the peaks. The time between the peaks of the parabolas is used to define the delay τ.

Here, we propose a different approach to determine temporal delays. Instead of computing (Equation 2) of the entire BOLD time series, we make use of the fact that the BOLD-triggered events have a well-defined timing (see Figure 3). Given a source time series *x*_*i*_(*t*) and a target time series *x*_*j*_(*t*), we obtain a set of *k*_*i*_ source events. For each source event in *x*_*i*_(*t*), we find the closest peak in *x*_*j*_(*t*) irrespective of its size and whether it occurred before or after the source event. We search for the peak within a window of [−6, 8] TRs from the source threshold crossing. As shown in Figure 3, to obtain a finer timing of both the source and target peak we also use a parabolic fit. The lag τ(*i, j*) is then defined as the difference between the timing of the target and the peaks of the source parabola. As a technical side note, when getting a peak value at the left or right edge of the time window we do not perform the parabola peak estimation, which could have unbounded values, but we set the lag to −6 or 6, respectively. If there is a particular interest, the same approach could be used to search for a negative peak (i.e., a de-activation) following a source event and estimate the activation de-activation delay between specific ROIs.

Since the sets of source (threshold crossing) events of *x*_*i*_(*t*) and *x*_*j*_(*t*) can be (and usually are) different, the matrix τ(*i, j*) is, in general, non-symmetric irrespective of the length of the time series. Additionally, for each *i, j* pair of ROIs we can obtain a set of delays for each individual source event *k*: τ^(*k*)^(*i, j*), an average of these values $\bar{\tau }\left(i,j\right)$, or alternatively a delay between average events τ(*i, j*) (like the ones in Figures 1D–F).

## 3. Results

In this section, we will proceed to describe the performance of the method. It will be carried out on two settings: The first (section 3.1) corresponds to the analysis of BOLD time series from 90 ROIs defined by the automated anatomical labelling (AAL) parcelation (Tzourio-Mazoyer et al., 2002), and the second (section 3.2) describes a voxel-wise functional connectivity analysis using both the classical Pearson correlation and our methodology. From the outset, we note that the objective of these comparisons is not to re-interpret or scrutinize the study under replication, but only to illustrate the use and caveats of our method. The validation of our method needs to wait for the use of this approach by others in different settings. To facilitate those enquires, the code is available at the repository https://github.com/remolek/NFC.

### 3.1. Functional Connectivity, Delay, and Directionality Computed From AAL Parceled Time Series

**Figure 4:**
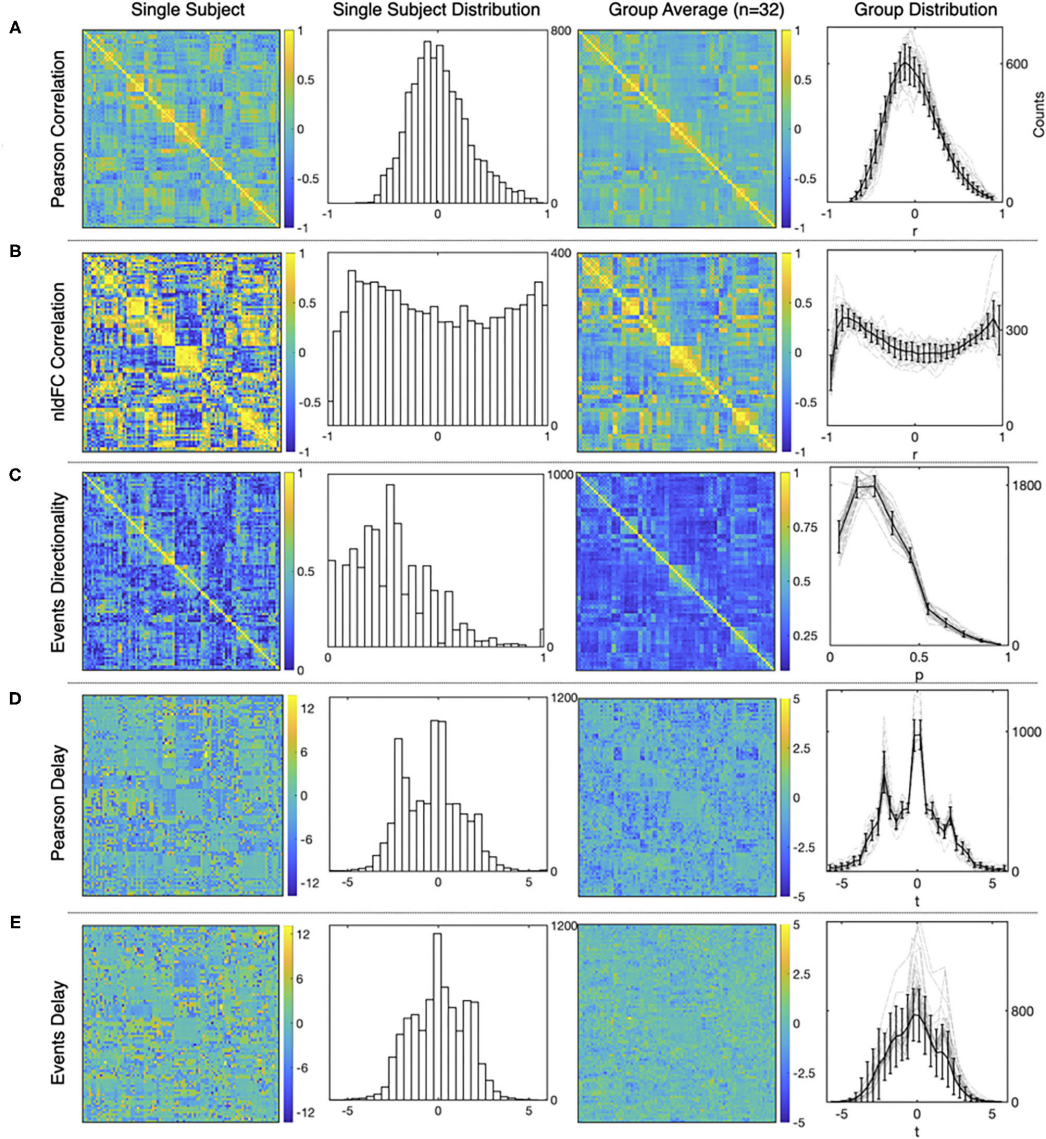
Examples of the matrices and distributions for each calculation performed over a fMRI dataset of 90 time series from the AAL atlas Tzourio-Mazoyer et al. (2002). The first and the second columns corresponds to a single subject statistics, while the average results from a group of healthy subjects (*n* = 32) are shown in the third and the fourth column. **(A–E)** show results for Pearson functional correlations, event correlations, event asymmetry, Pearson's delay, and event delay, respectively. For each measure, the first and the third columns show results in a matrix format, while the second and the fourth columns show the distributions of each measure (mean values and S.D. error bars are used for the group distributions).

Here, we will provide examples of typical results of the computations explained previously. To that aim, we will use fMRI BOLD data from 32 healthy participants downloaded from the Autism Brain Imaging Data Exchange (ABIDE) database (Craddock et al., 2013). Each dataset comprises 90 AAL preprocessed time series (using Data Processing Assistant for Resting-State fMRI (DPARSF) pipeline). In all cases, the time series are demeaned and normalized to their SD (i.e., z-scored),

Typical results from the computations using both, the standard FC approach and our method are presented in Figure 4. For each of the three measures and for both methods, the figure shows a matrix from single subject results, a mean matrix of the whole group and the distributions for each of the computations.

First, Figure 4A shows typical results obtained from Pearson's correlations between all the time series, and note that the distribution exhibits the usual Gaussian shape. This is not the case for the distribution of event correlations (Figure 4B) that is expected for the sampling distribution of Pearson's estimator for a small length of time series. This feature is further discussed in Supplementary Material 3.

Figure 4C shows the matrix and the distribution of the edges' directionality computed as the proportion of shared events between regions (two leftmost panels) as explained in section 2.3. The alternative measure performed by subtracting the transposed matrix is shown in Supplementary Material 3.

Delay between time series is shown in Figure 4D, for shifted time series as in Mitra and Raichle (2018) and Figure 4E, for delay computed using events. Note that for Figure 4D, the apparent asymmetry is due to the TRs subtracted at the beginning and end of the signal, to allow the computation, while for Figure 4E, the event selection between target and source, so it is not an artifact of the computation.

To further inspect the behavior of these metrics, we computed average path length and clustering coefficient of the networks given a certain threshold, and it can be seen in Supplementary Material 2.

### 3.2. Replication of Voxel-Wise Functional Connectivity Findings

As a further test of the computations explained above, we have used fMRI data from the ABIDE preprocessed database (Craddock et al., 2013) to replicate recent findings on functional connectivity between insular sub regions on Autism Syndrome patients Xu et al. (2018). ABIDE is an open database with thousands of pre-processed fMRI brain scans of Autistic Syndrome patients (AU) and age-matched Healthy subjects (HS) http://preprocessed-connectomes-project.org/abide/quality_assessment.html (Rolls et al., 2016; Zheng et al., 2016; Dadi et al., 2019). For these computations, we collected a sample of 47 AU and 32 HS. The MRI data acquisition as the preprocessing pipeline used can be accessed here: http://preprocessed-connectomes-project.org/abide/Pipelines.html.

#### 3.2.1. Pearson's Correlations

**Figure 5:**
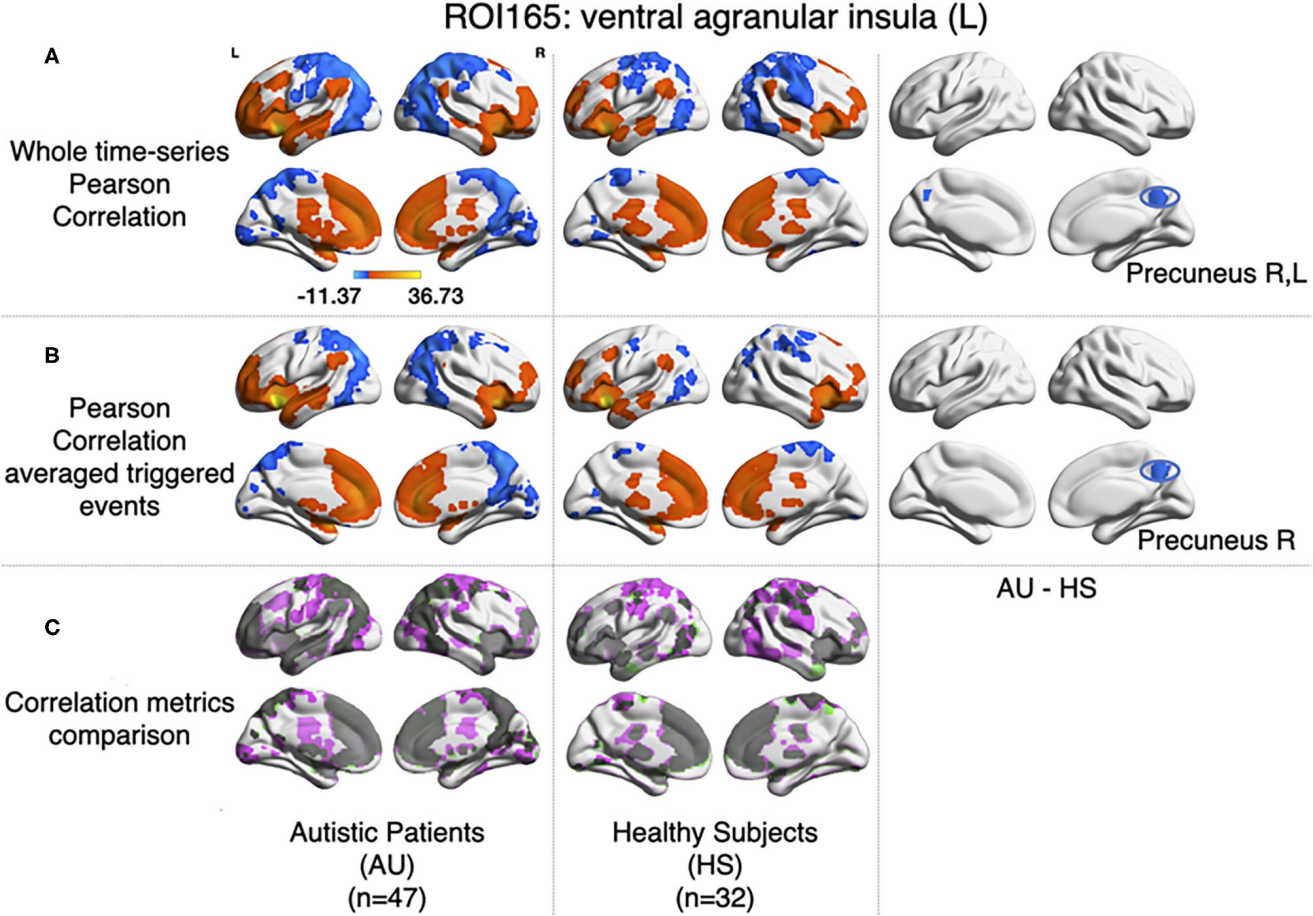
Comparison between Pearson's correlation of the BOLD time series and the correlation of the large amplitude BOLD events. **(A)** Results for zero-lag seed-voxel correlations using the left ventral agranular insula as seed. It is shown the Z-transformed Pearson's correlation one-sample *t*-test for each group of participants (first-second columns for AU and third-fourth for HS) and two-sample *t*-test to assess differences between groups (fifth and sixth columns of brain surfaces) (GFR corrected voxel *p* < 0.001, cluster *p* < 0.05). **(B)** The same distribution of columns as **(A)**, for the results of correlating the large events (Z-transformed, GFR corrected voxel *p* < 0.001, cluster *p* < 0.05). **(C)** Correlations from A and B compared, gray areas show coincidences between metrics, pink shows correlations only detected in A and green correlations only detected in **(B)**.

For each subject, the average BOLD time series from six insular subregions (using brainnetome functional atlas, Fan et al., 2016) as extracted and correlated using Pearson's correlation with all the rest of the voxels of the brain (gray matter masked) as in Xu et al. (2018). One-sample *t*-test was computed for each group of participants (AU and HS) to result in the correlation pattern of each insular subregion, obtaining comparable results as in Xu et al. (2018) (Figure 1). In this study, we are showing results from the left ventral agranular insula subregion as a proof of replication. As in Xu et al. (2018), HS resulted in higher correlation of this ROI with bilateral precuneus cortex (see Figure 5).

#### 3.2.2. Nonlinear Functional Co-activations

Following the method explained in Tagliazucchi et al. (2011), relevant events from the mean time series of left ventral agranular insula subregion were extracted (triggering events where the amplitude is above a 1 S.D. threshold, 2 TR previous to this trigger, and 4 TR after). All time series from all the voxels in the brain (gray matter masking) corresponding to those events were extracted. Then, the correlations between the average source event of the insula and the average target event of each voxel were computed. As it can be observed in Figure 5B, similar results to Pearson's correlation of the whole signal were obtained (note that here we have only taken into account the signal from events, not the whole time series). The same cluster of higher correlation between insula and precuneus cortex in the HS group can be observed by computing a two-sample *t*-test (GFR corrected, p-voxel = 0.001, p-cluster = 0.05).

#### 3.2.3. Directionality

**Figure 6:**
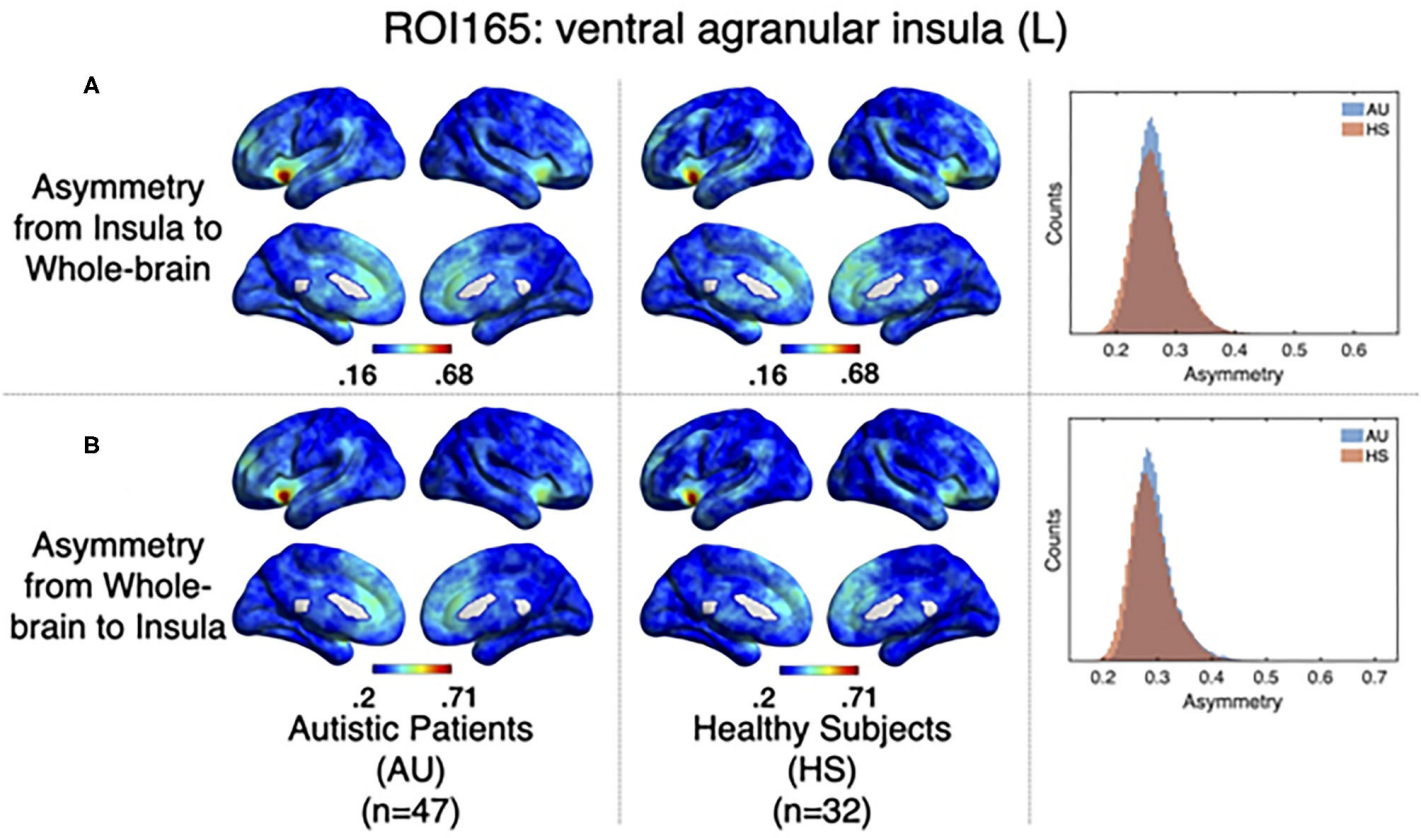
Directionality computed from-insula and to-insula for all brain voxels. **(A)** Shows group averages for asymmetry from insula subregion mean time series to the rest of the brain for Autistic patients (left columns), Healthy subjects (middle columns), and histograms showing the distribution of these directionalities. **(B)** Shows the same computations but for directionality from all the brain to the Insula subregion mean time series.

**Figure 7:**
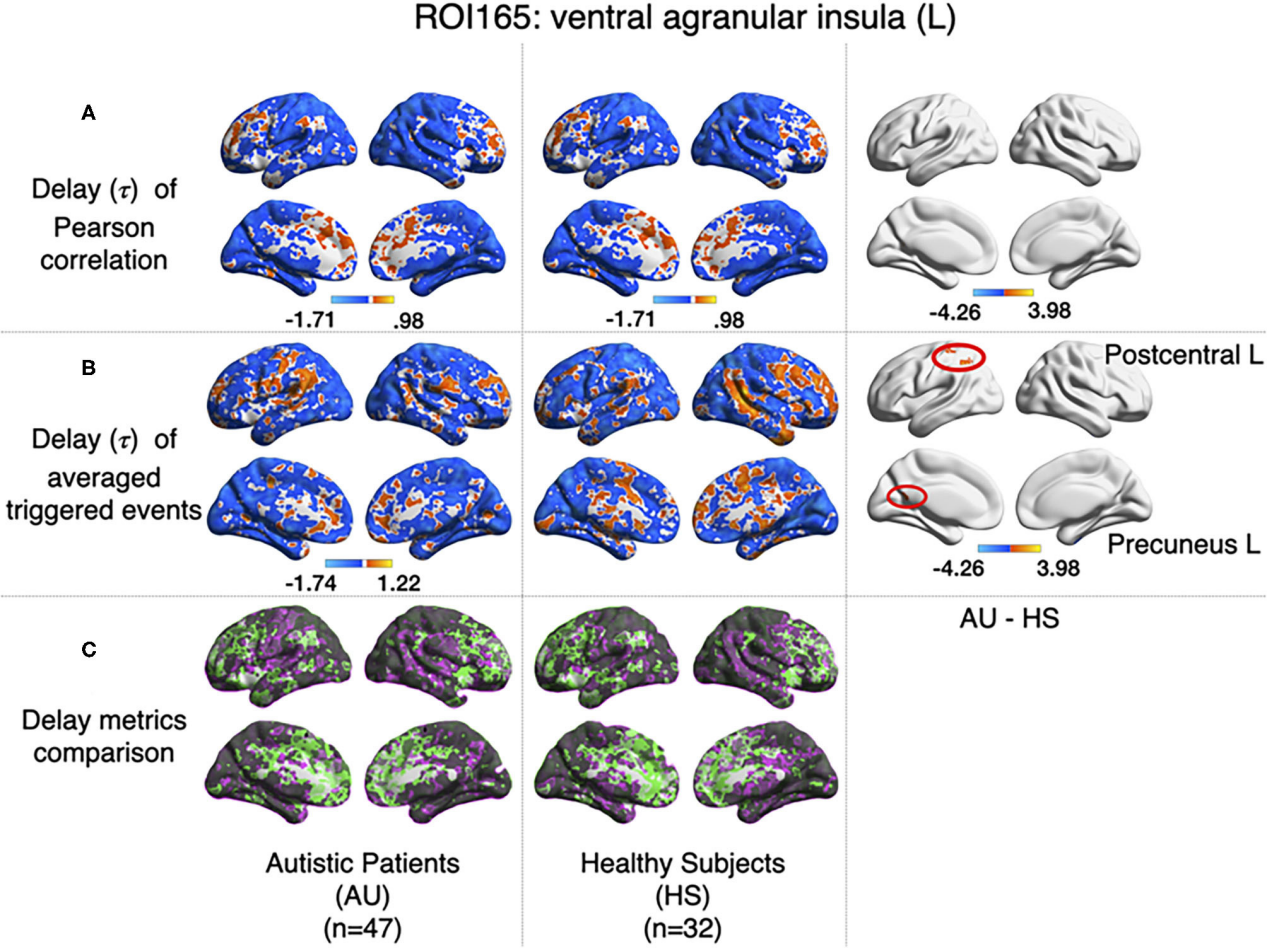
Comparison of the delays values estimated with the Pearson **(A)** and the event correlation **(B)** for AU and HS. Left columns show the average delays for AU, middle columns the average delays for HS, and the right column the comparison between groups (AU minus HS). The two circles (postcentral gyrus) indicate significant differences not evident with standard methods (GFR corrected voxel *p* < 0.001, cluster *p* < 0.05). **(C)** Compares **(A,B)**, where gray areas show coincidences between metrics; pink shows areas where B>A, and green shows A>B.

As it has been explained above, the correlation value between two signals (i,j) obtained when computing relevant events is not symmetric. The correlation of the source events with its target *r*(*i, j*) is not necessarily the same as the correlation of the events of that target, acting as a source, with the original source, acting as a target *r*(*j, i*). The difference between this *r*(*i, j*) and *r*(*j, i*) can be understood in terms of directionality of the correlations. To test whether the functional activity of the left ventral agranular insula exhibits such property, we computed directionality across the whole brain. Overall, we have observed only very small differences (see histograms in Figure 6) but they are no significant differences between groups in specific areas (Figure 6, GFR corrected all *p* > 0.05). This contrasted with the significant findings we found for the correlation and delay computations (Figures 5, 7. The density distributions shown in Figure 6 (right panels) indicate that in both, HS and AU subjects, the correlations are directed (asymmetric) and that the mode of the directionality is most frequent in the AU subjects (depicted in light blue) than in the HS (*p* < 0.01).

#### 3.2.4. Delay

**Figure 8:**
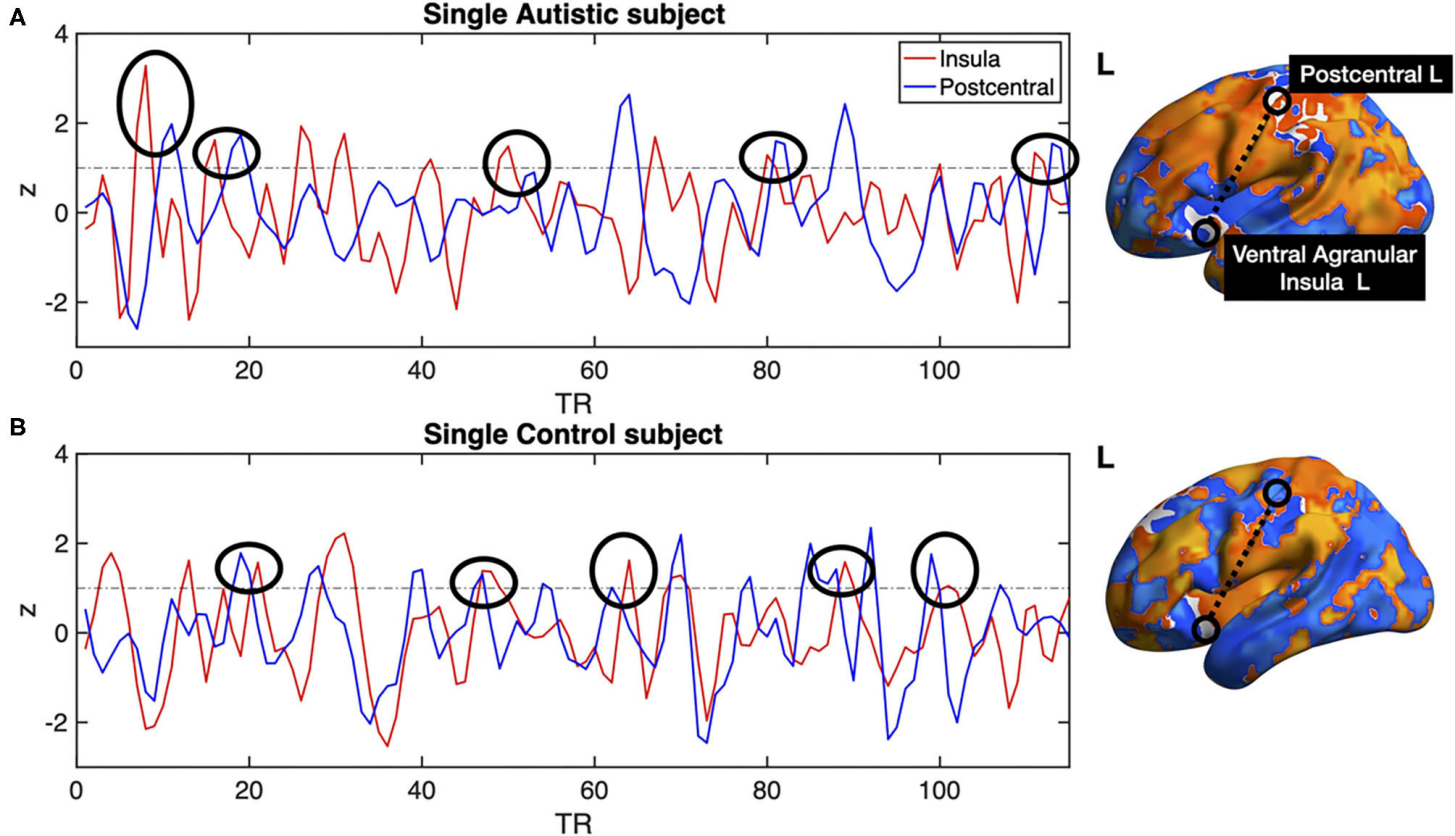
Single subject postcentral gyrus (blue lines) and ventral agranular insula (red lines) BOLD time series. **(A)** shows example data from an AU, **(B)** shows data from a HS. Black circles show several instances in which the delays between the time series are consistent with the statistics compiled from the entire groups shown in Figure 7. The example shows that the insular events on the AU patient are usually preceding the postcentral events, resulting in a negative delay, while the opposite results are observed on the BOLD time series of the Healthy Subject.

All previous computations correspond to correlations computed at equal time. In addition, it is straightforward to estimate the average delay between the peak of the source events to the peak of its closest target events. We computed this delay measure from the source events extracted from the left ventral agranular insula in respect to all the rest of the brain voxels. Comparing the delays between the groups, it can be seen that while the left postcentral gyrus and the precuneus cortex exhibit a positive delay in the AU group, the HS subjects show a negative delay (Figure 7). To illustrate these delay differences, Figure 8 shows examples of time series of the postcentral gyrus and the ventral agranular insula for a single AU subject (Figure 8A) and an HS subject (Figure 8B).

## 4. Discussion: Features, Advantages, and Limitations of the Proposed Strategy

Since its introduction, almost a decade ago, it has been suggested that the point process (or its variants) extracted from the large amplitude BOLD deflections contains enough dynamical information (Tagliazucchi et al., 2011, 2012), to identify the timing and the location of epochs of high correlations among brain regions. This identification has acquired relevance in the context of dynamical functional connectivity see, for instance, the reviews by (Keilholz et al., 2017) and (Iraji et al., 2020). In line with this, the recent report of Esfahlani et al. (2020) emphasizes the fact that few events of co-activation can estimate the functional connectivity architecture of a system, a finding that is in full agreement with our original arguments. Thus, it is important to remark that behind all these reports there is a basic reason why these few points contain most of the information as discussed recently (Cifre et al., 2020).

Emphasizing the relevance of relatively high amplitude BOLD signal while compressing the data motivated the two paradigms we have proposed previously, namely, the point process (Tagliazucchi et al., 2012) and the so-called rBeta technique (Tagliazucchi et al., 2011). Both attempt to capture the spatio-temporal dynamics with the smallest possible sampling with a trade-off between temporal and spatial resolution. The point process compresses in the temporal domain, which implies that to smoothly represent spatio-temporal correlated patterns, one needs to sample more voxels. On the other hand, the rBeta approach uses much fewer voxels, but at the expense of keeping additional temporal information around each threshold crossing. These two variants have demonstrated two main advantages comparing to the above-mentioned functional connectivity measures, the first one is that they imply a data size reduction and less computational resources to obtain comparable results to full time series analyses, and second, as these are only focusing on relevant high amplitude time-points, or events, non-significant events occurring during the scan are not blurring the computations.

It is important to remind that, in terms of neurophysiology, the observed changes in the BOLD signal can not be simply and exclusively attributed to change in neural responses (Aguirre et al., 1998; Noseworthy et al., 2003; Handwerker et al., 2004; Raut et al., 2021). The HRF variability has been pointed several times as a common confounder in the determination of functional connectivity using Pearson's correlation of the BOLD signals (Rangaprakash et al., 2018; Yan et al., 2018). It has been suggested the need to de-convolve the BOLD signal in order to obtain a confounder-free robust FC [as discussed in Wu et al. (2013) and more recently in Wang et al. (2020)].

In that regard, the present approach explicitly takes into account such variability because the source events extracted from any given ROI represents (by construction) the *local* HRF. This similarity was already noted in Tagliazucchi et al. (2012) by comparing the de-convolved BOLD signal using either a canonical HRF or the source event extracted by our approach (see Figure 1D in Tagliazucchi et al., 2012). The most recent work of Urunuela et al. (2021) summarizes this point very well: “deconvolution approaches hold a close parallelism to recent methodologies aiming to understand the dynamics of neuronal activations and interactions at short temporal resolution and that focus on extreme events of the fMRI signal (Lindquist et al., 2007).” In that work, the authors provide a very persuading evidence of such parallelism: “Figure 6 shows that the innovation- or activity-inducing CAPs computed from deconvolved events in a single resting-state fMRI dataset closely resemble the conventional CAPs computed directly from extreme events of the fMRI signal (Tagliazucchi et al., 2011, 2012, 2016; Liu and Duyn, 2013; Liu et al., 2013, 2021; Cifre et al., 2020; Zhang et al., 2020; Rolls et al., 2021).”

The nonlinear dynamic functional connectivity method we are proposing offers an unexplored and widely different perspective in the analysis of brain co-activation patterns without much numerical complications, since it implies no more than thresholding and the computation of linear correlations, facilitating a simple interpretation of the resulting functional connectivity paths. The fact that the correlations are computed from events identified either as sources or targets allows for a straightforward definition of directed graphs (i.e., asymmetric correlation matrices). These source-target relations may lead to novel approaches to understand brain dynamics, for instance, as in the example of Autism Syndrome in which the computation of delays between events showed uncovered distinct information. Indeed, the Pearson correlation, computed between left ventral agranular insula and postcentral gyrus, does not show any differences between AU and HS, while it has been reported that postcentral gyrus has a differential connectivity in Autistic Syndrome when analyzing big samples Gu et al. (2015). However, when we computed the delays from insula to other regions, differences between the two groups in Postcentral Gyrus appeared, which leads us to think that this differential connectivity may be expressed on a spatio-temporal domain. Another example is the additional difference we have found between the two groups concerning a weaker functional connectivity between precuneus cortex and ventral agranular insula, which is accompanied by the above-mentioned differences in delay (Figure 7B).

Note two practical advantages provided by the present approach. The results are highly reproducible on correlations asymmetry and delays, being robust to changes in the threshold used to extract the source events. The method is equally applicable to the analysis of fMRI data during a task, by extracting the source events from the task convolved with a HRF function. A similar approach can be used to study dynamic functional connectivity fluctuations possibly due to ongoing cognition, as suggested in Gonzalez-Castillo et al. (2014).

Further testing of the method should be performed to identify more specifically its limitations. For instance, we have not compared the method with results obtained from sliding-window Pearson's correlation, a widely used method to inspect dynamics in functional connectivity (Hutchison et al., 2013; Preti et al., 2017). In further work, we expect that will uncover a relation between this window-based functional connectivity and the information provided from the delays of our method. Another point that deserves to be clarified is the meaning of the peaks in the delay distribution, something already intriguing from previous results obtained using Pearson's correlation delays (see Figure 5 in Mitra et al., 2015a), which was recently related to very slow arousal fluctuations (Raut et al., 2021).

Finally, we shall mention that while here we concentrated on the activation events, i.e., denoted by the BOLD signal upward crossing of a threshold, the same method can be applied without modification to *de-activation* events. In such a way, graphs of regions of interest to are correlated with the deactivation of regions can be obtained, something that we are not aware was considered before.

In conclusion, we have analyzed undisclosed properties of the previously published rBeta method (Tagliazucchi et al., 2011). Overall, these calculations provide a different kind of information than the usual Pearson correlation of the entire BOLD time series. As a proof of concept, we have used the method to replicate a recently published study of functional connectivity in Autism Syndrome, reproducing their main findings and uncovering additional features. Given that the proposed approach implementation is simple and robust, it is expected that future work can be dedicated to validate and extend the method to other settings and experimental paradigms.

## Data Availability Statement

Publicly available datasets were analyzed in this study. This data can be found here: http://adni.loni.usc.edu/.

## Author Contributions

IC, MTM, LP, JO, and DC: contributed on manuscript writing process. IC, LP, JO, and DC: contributed on reviewing the manuscript, results discussion, and data analysis. All authors contributed to the article and approved the submitted version.

## Funding

This work was supported by the MICINN (Spain) grant PSI2017-82397-R, the National Science Centre (Poland) grant DEC-2015/17/D/ST2/03492 and the Foundation for Polish Science (FNP) project Bio-inspired Artificial Neural Networks grant POIR.04.04.00-00-14DE/18-00, and by CONICET (Argentina) and Escuela de Ciencia y Tecnología, UNSAM. Work conducted under the auspice of the Jagiellonian University-UNSAM Cooperation Agreement.

## Conflict of Interest

The authors declare that the research was conducted in the absence of any commercial or financial relationships that could be construed as a potential conflict of interest.

## Publisher's Note

All claims expressed in this article are solely those of the authors and do not necessarily represent those of their affiliated organizations, or those of the publisher, the editors and the reviewers. Any product that may be evaluated in this article, or claim that may be made by its manufacturer, is not guaranteed or endorsed by the publisher.
