# Supplementary material for: Revisiting Nonlinear Functional Brain Co-activations: Directed, Dynamic, and Delayed
Source: Front Neurosci. 2021 Oct 12;15:700171. doi: 10.3389/fnins.2021.700171 (PMC8546168; doi:10.3389/fnins.2021.700171)
Supplement: Supplementary file 1 [file Image_1.pdf]

Supplemental material for: "Revisiting non-linear functional brain co-activations: directed, dynamic and delayed" by Cifre *et al.*

1. CORRELATION ASYMMETRY

As explained in section 2.3, the degree of asymmetry in the correlation between pairs of ROI's can be estimated in two ways. The results presented in the main text were computed by estimating the relative number of events (see Section 2.3). Here, we present an example of the computation by the other approach, which is subtracting the event correlation matrix from its transposed matrix.

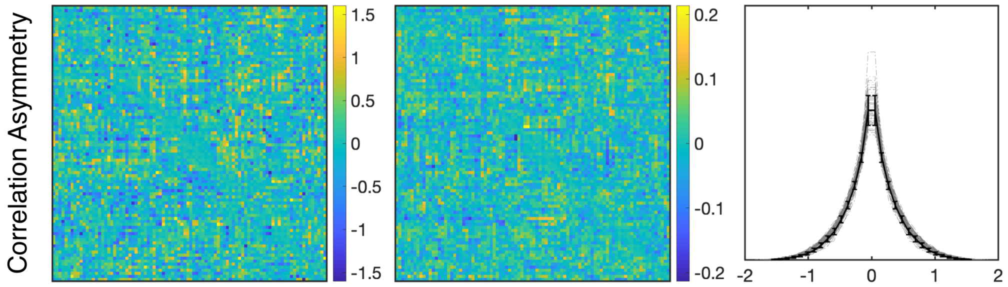

FIG. S1. Asymmetry computed from subtracting event's correlation matrix to its transposed matrix. Matrix on the left is a single subject example, matrix in the middle is the average across all subjects (n=32) and the right plot is the distribution of this computation (grey dashed lines are the distribution for each subject and black line is the averaged distribution (error bars are S.D.)).

2. NETWORKS

In Fig.S2 examples of networks constructed with the quantities defined in Fig. 4 are presented. The time series are taken from 90 AAL regions [6]. The left column represents the distribution of values (either correlation, directionality or delay) used to construct the networks. The right column shows the clustering coefficient and the path length computed for each network as a function of the threshold used to define the presence or not of a given edge. Eventually our approach can be used to define multilayer networks [1, 5] where one level for correlation, other for delay and another for directionality.

3. STATISTICAL SIGNIFICANCE: SAMPLING DISTRIBUTION OF CORRELATIONS

In this section, we briefly clarify what distributions of triggered event correlations can be expected. It only applies to long time averages, and do not include non-stationary changes in time series.

Given two time series s_i and s_j (or, in our case, two BOLD triggered events) of length T, Pearson estimator r of linear correlation coefficient is given by

$$r_{i,j} = \frac{\sum_{t=1}^{T}(s_{it} - \bar{s}_i)(s_{jt} - \bar{s}_j)}{\sqrt{\sum_{t=1}^{T}(s_{it} - \bar{s}_i)^2}\sqrt{\sum_{t=1}^{T}(s_{jt} - \bar{s}_j)^2}} \tag{1}$$

where $\bar{s}_i = \frac{1}{T}\sum_{t=1}^{T} s_{it}$ is the sample mean and σ_i sample standard deviations of the event s_i.

One assumes that there is a true linear correlation coefficient ρ that corresponds to a very long time series of such events. It is known that such an estimator of ρ for two Gaussian correlated series is biased, and that the sampling distribution of correlation coefficients r is given by [4]

$$P(r) = \frac{(T-2)\left(1-\rho^2\right)^{\frac{T-1}{2}}\left(1-r^2\right)^{\frac{T-4}{2}}\Gamma(T-1)}{\sqrt{2\pi}\Gamma\left(T-\frac{1}{2}\right)(1-\rho r)^{T-\frac{3}{2}}}\,{}_2F_1\left(\frac{1}{2},\frac{1}{2};\frac{2T-1}{2};\frac{r\rho+1}{2}\right), \tag{2}$$

where ρ is the population correlation coefficient, ${}_2F_1(a,b;c;x)$ is a hypergeometric function, and $\Gamma(z)$ is the gamma function.

As can be observed in Fig. S3 A, for non-zero ρ the distribution of Pearson correlation coefficient r is biased. The mode of the distribution for $\rho > 0$ is further to the right than the true correlation. Indeed we observe a similar skewed distribution for triggered events, as can be seen in Fig.4B, while linear correlation estimates are less skewed due to much larger time series length T.

Since the BOLD signals are not normally distributed, and are strongly auto-correlated, we fitted $P(r)$ to obtain an estimate of the length T of Gaussian time series that would produce approximately the same distribution. In Fig. S3 B, we show such a fit to a histogram of BOLD $18s$ long signal events. The events are randomly sampled in order to obtain a sampling distribution for $\rho = 0$. The resulting estimate is $T = 4.51 \pm 0.02$.

Further, to confirm that the distributions (2) can be generated by auto-correlated series, we simulated ARMA(2,2) models. The results, shown in Fig. S3 C, closely resemble the

theoretical expectation. Additionally, we show an example of how adding two weighted distributions (2) with positive and negative ρ can results in a distribution similar to the histogram in Fig.4B.

The correlation estimate can be simply corrected by a Fisher transform [2, 3]. It is also possible to find a maximum-likelihood estimate of ρ, knowing the form of the distribution (2). Another option is calculating $\hat{\rho} = r\,(1 - (1 - r^2)/2T)$ as suggested in [4]. Lastly, good results are also obtained by simply taking an average of r estimates (as shown in Fig. S3 A and C).

REFERENCES

[1] De Domenico, M., Solé-Ribalta, A., Cozzo, E., Kivelä, M., Moreno, Y., Porter, M., Gómez, S., Arenas, Alex Mathematical formulation of multilayer networks. *Physical Review X* 4, 1–15. doi:10.1103/PhysRevX.3.041022

[2] Fisher, R.A., On the probable error of a coefficient of correlation deduced from a small sample, *Metron*, 1, 3-32 (1921).

[3] Fisher, R.A. , Frequency Distribution of the Values of the Correlation Coefficient in Samples from an Indefinitely Large Population, *Biometrika*, 10(4), 507-521 (1915).

[4] Kenney, J.F., & Keeping, E.S., Mathematics of statistics, part two, Princeton, NJ: Van Nostrand Company, 217-221 (1951).

[5] Kivela, M., Arenas, A., Barthelemy, M., Gleeson, J.P., Moreno, Y., Porter, M.A. Multilayer networks. *Journal of Complex Networks* 34, 203–271. doi:10.1093/comnet/cnu016

[6] Tzourio-Mazoyer, N., Landeau, B., Papathanassiou, D., Crivello, F., Etard, O., Delcroix, N., et al. (2002). Automated Anatomical Labeling of Activations in SPM Using a Macroscopic Anatomical Parcellation of the MNI MRI Single-Subject Brain. *Neuroimage* 15, 273–289. doi: 10.1006/NIMG.2001.0978

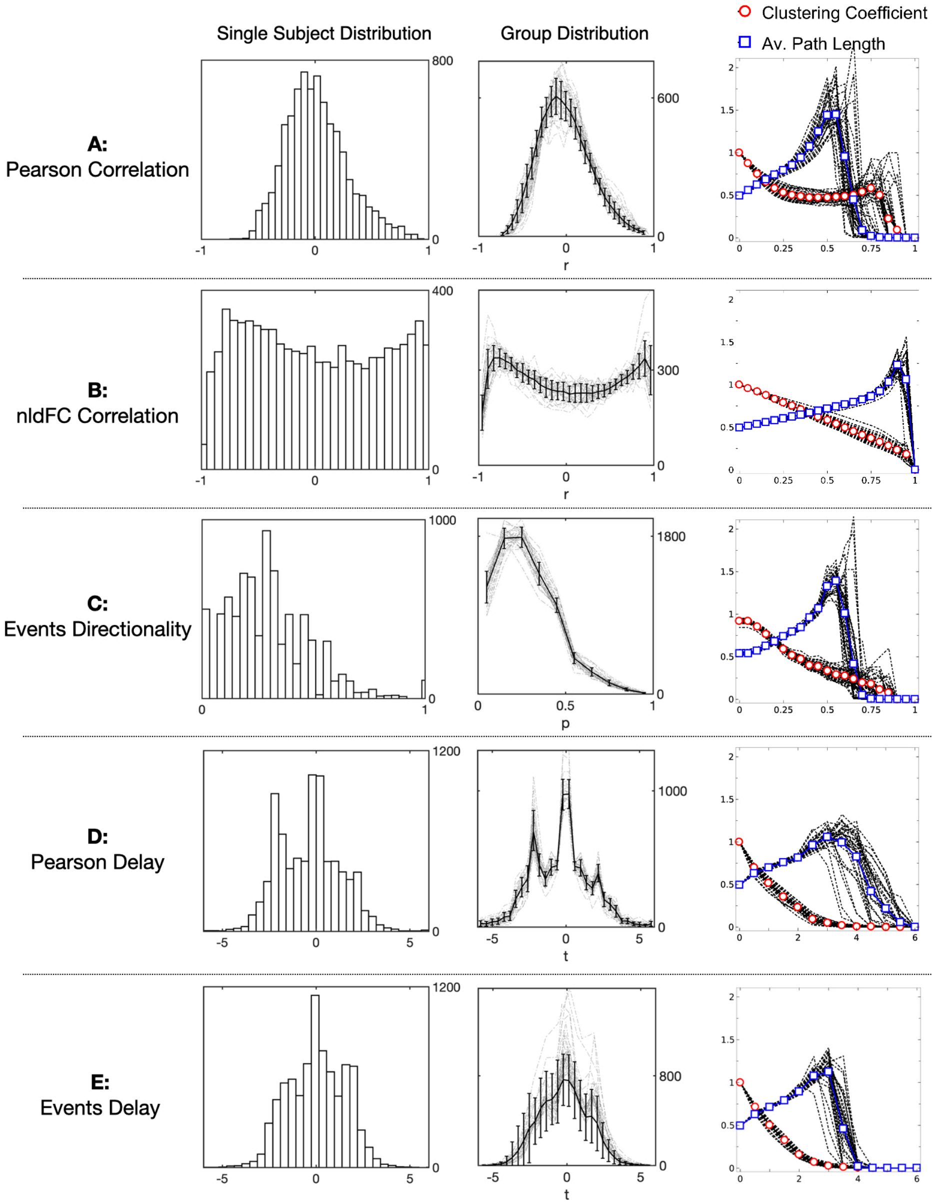

FIG. S2. Complex functional brain networks can be constructed from the current approach. The two leftmost panels depict the statistics already presented in Figure 4 for both the single subject and group distributions. The right panels show the global clustering coefficient (red circles) and the average path length (blue squares) of binary networks constructed from these quantities as a function of the threshold (on the horizontal axis). Black lines are results for single control subjects; circles and squares correspond to averages from the entire group (n=32).The adjacency matrices are binary, made by thresholding the correlation (or directionality, or delay) matrices. In the case of correlations and delays, absolute value was taken. When the network became disconnected (around the visible peak of the clustering coefficient in the plots), the average path length was computed for the largest connected component. Each network is computed from 90 timeseries extracted from the AAL defined regions [6].

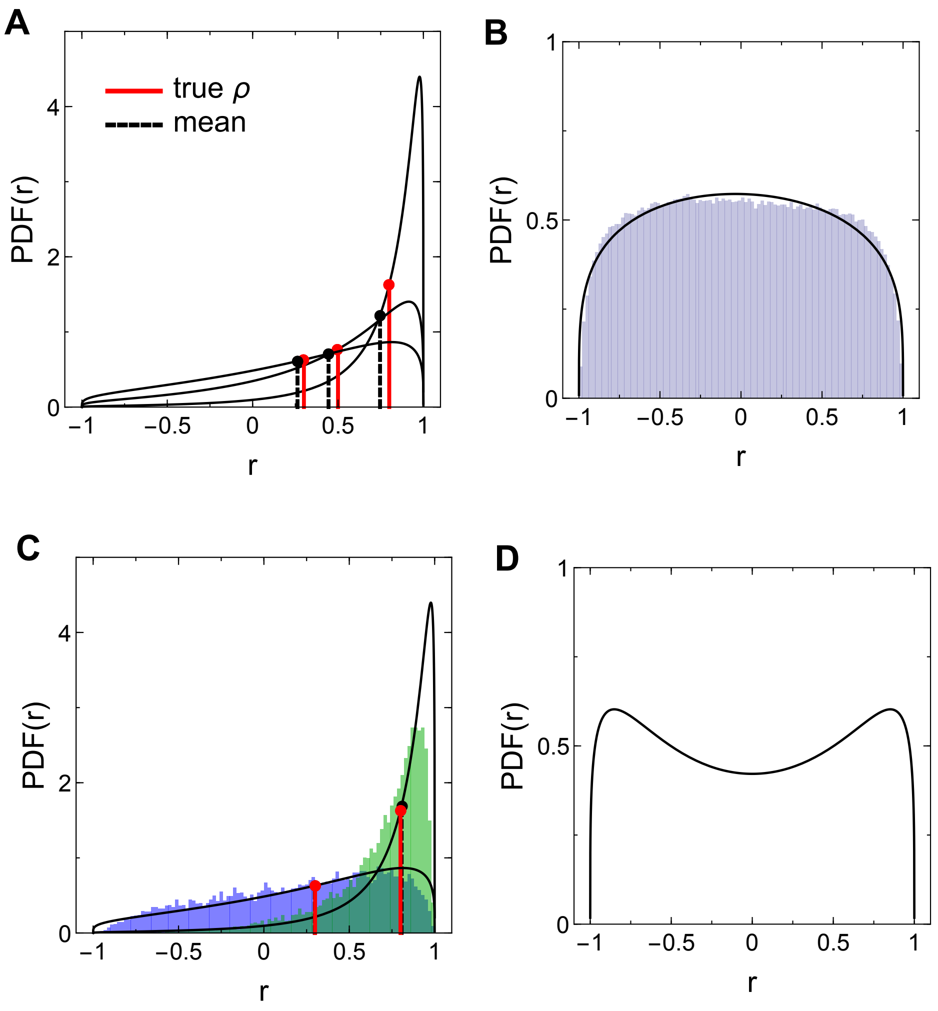

FIG. S3. Sampling distributions of short time series. (A) sampling distributions (2) of Pearson estimator for small $T = 4.5$ and $\rho = 0.3, 0.5, 0.8$ (vertical red lines), together with the distribution means (vertical black dashed lines). (B) Fit of distribution (2) to randomized event correlations, showing that $T = 4.5$ for corresponds to auto-correlated BOLD event of $18s$ length. (C) Histograms of Pearson correlations obtained for pairs of simulated series with a predefined auto-correlation and true linear cross-correlation $\rho = 0.3, 0.8$. The true ρ and the histogram distribution average coincide (red and dashed vertical lines). (D) An example of adding two equally weighted distributions (2) with $\rho = 0.4$ and -0.4, similar to the histogram in Fig.4B.
